# Utility of Induced Pluripotent Stem Cells for the Study and Treatment of Genetic Diseases: Focus on Childhood Neurological Disorders

**DOI:** 10.3389/fnmol.2016.00078

**Published:** 2016-09-06

**Authors:** Serena Barral, Manju A. Kurian

**Affiliations:** ^1^Neurogenetics Group, Molecular Neurosciences, UCL Institute of Child Health,University College LondonLondon, UK; ^2^Department of Neurology, Great Ormond Street HospitalLondon, UK

**Keywords:** iPSCs, childhood neurological disorders, *in vitro* disease modeling, gene therapies, drug screening, isogenic control

## Abstract

The study of neurological disorders often presents with significant challenges due to the inaccessibility of human neuronal cells for further investigation. Advances in cellular reprogramming techniques, have however provided a new source of human cells for laboratory-based research. Patient-derived induced pluripotent stem cells (iPSCs) can now be robustly differentiated into specific neural subtypes, including dopaminergic, inhibitory GABAergic, motorneurons and cortical neurons. These neurons can then be utilized for *in vitro* studies to elucidate molecular causes underpinning neurological disease. Although human iPSC-derived neuronal models are increasingly regarded as a useful tool in cell biology, there are a number of limitations, including the relatively early, fetal stage of differentiated cells and the mainly two dimensional, simple nature of the *in vitro* system. Furthermore, clonal variation is a well-described phenomenon in iPSC lines. In order to account for this, robust baseline data from multiple control lines is necessary to determine whether a particular gene defect leads to a specific cellular phenotype. Over the last few years patient-derived neural cells have proven very useful in addressing several mechanistic questions related to central nervous system diseases, including early-onset neurological disorders of childhood. Many studies report the clinical utility of human-derived neural cells for testing known drugs with repurposing potential, novel compounds and gene therapies, which then can be translated to clinical reality. iPSCs derived neural cells, therefore provide great promise and potential to gain insight into, and treat early-onset neurological disorders.

## Introduction

Over the last decade, significant advances such as whole exome and genome sequencing have facilitated genetic screening of patients, resulting in an ever-increasing number of inherited human diseases. Despite this genetic revolution, the molecular mechanisms downstream of a specific gene mutation or genetic variant remain yet to be fully elucidated for the majority of diseases. Future research priorities must therefore lie in studying such disorders in more depth, to not only understand the disease, but also to develop novel therapies for clinical translation.

To date, transgenic animal models and transformed cell lines, have allowed clarification of pathophysiological pathways affected by genetic mutations. Despite their benefits, both methods have a number of limitations, in that they often do not fully mimic human physiology, only partially recapitulate progression of disease, and do not accurately recapitulate human metabolism and homeostasis. It has long been recognized that patient derived cells are a potentially better *in vitro* tool for studying human disease. However, human tissue is often either unavailable or simply not accessible. This is clearly exemplified by neurological disorders, where accessing the brain and neuronal tissue for cell culture and future study is near impossible. Since the first human embryonic stem cells (ESCs) were isolated in 1998 (Thomson et al., 1998), the use of pluripotent stem cells (PSCs) has become a new reality in the study of human diseases, offering a challenging but incredibly useful model to move from clinic to bench and potentially vice versa.

The discovery of cellular reprogramming techniques has been a major step forward in the *in vitro* modeling of human disease, theoretically allowing the study of all genetic disease with specific patient cells as the starting point. Yamanaka and colleagues (Takahashi et al., 2007) elegantly reprogrammed adult dermal fibroblasts to a pluripotent state, by inducing ectopic expression of four factors: Oct4, Sox2, Klf4, and cMyc (Takahashi et al., 2007; Takahashi and Yamanaka, 2016). The induced PSCs generated are highly similar to human ESCs, with the ability to indefinitely proliferate and differentiate in cells derived from the three germ layers. Since publication of Yamanaka’s landmark article, reprogramming techniques have been further refined, and many new strategies have been developed to effectively reprogram somatic cells into pluripotency. Integrating retroviruses and lentiviruses have been superseded by the use of non-integrating systems, including adenovirus, Sendai virus, mRNA, episomal vectors, proteins and small molecules (Fusaki et al., 2009; Kim et al., 2009; Zhou and Freed, 2009; Warren et al., 2010; Okita et al., 2011; Bar-Nur et al., 2014).

**Figure 1:**
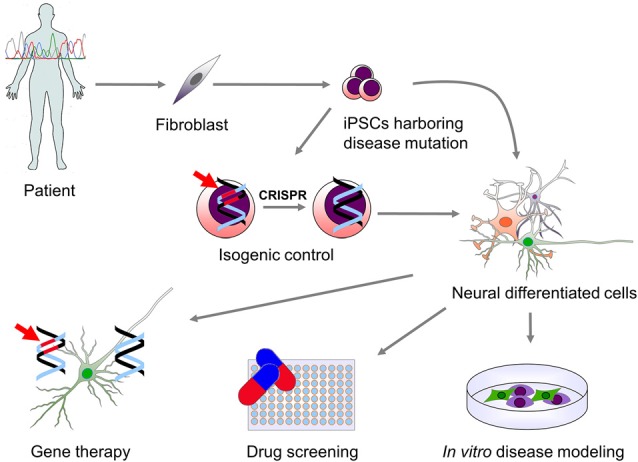
**Use of patient-derived induced pluripotent stem cells (iPSCs) for modeling genetic neurological diseases.** Genetic screening of patients affected by a neurological disorder may lead to the identification of specific mutations causing disease. Patient-derived somatic cells (fibroblast and other cell types) can be then reprogrammed to a pluripotent state. iPSCs carrying the disease-related mutation (indicated in red) can be then differentiated into the neural cells type (neurons and glial cells) which are affected in the disease. This allows for the *in vitro* study of the molecular mechanisms downstream the genetic mutation. In order to overcome genetic background variability and to validate the effect of the genetic mutation on phenotype observed *in vitro*, isogenic control iPSCs can be generated via genomic correction of the mutation. Moreover, *in vitro* differentiated cells can be used for high-throughput screening of drugs or the validation of specific genetic therapies that can then be translated into clinical practice.

Neural stem cells (NSCs) have been successfully derived from PSCs and several protocols for PSCs differentiation into a broad variety of mature neurons and glial cell subtypes have been published (Srikanth and Young-Pearse, 2014). Patient-derived neural cells have the specific advantage of retaining the genetic background of the donor and thus offer a unique *in vitro* neuronal disease model. They are an unlimited source of cells that allows the analysis of the cellular mechanisms involved in disease. Furthermore, they provide a novel platform to test new drugs and genetic therapies as well as a source of cells that could potentially be used for cell replacement therapy (Figure 1).

When approaching the study of neurological diseases using human induced pluripotent stem cell (iPSC)-derived neurons as an *in vitro* model system, several considerations need to be taken into account, including the accurate generation of truly pluripotent cells, the relative efficiency of neuronal differentiation, and the strengths, utility and limitations of generated neurons. In this review, we provide a brief overview of what we consider to be the most important advantages and disadvantages of using human iPSCs to model neurological diseases, and their translational utility at a clinical level. Our main focus will be to evaluate this model system for early-onset genetic neurological disorders (Table 1), although, where relevant and appropriate, we will use examples from other later-onset neurological diseases.

**Table 1:** **Utility of induced pluripotent stem cells (iPSC) in childhood-onset neurodevelopmental and neurological disorders**.

Disease	Gene(s)	Differentiated cell type	Molecular characterization	Compound screening	Gene/RNA therapy
**Neurodevelopmental disorders**					
Rett syndrome	*MEPC2* *CDKL5*	Neural progenitor cells Neurons (glutamatergic) Astrocytes	Muotri et al. (2010), Amenduni et al. (2011), Ananiev et al. (2011), Cheung et al. (2011), Kim et al. (2011), Farra et al. (2012), Larimore et al. (2013), Williams et al. (2014), Andoh-Noda et al. (2015), Djuric et al. (2015), Fernandes et al. (2015), Livide et al. (2015), Tang et al. (2016) and Zhang et al. (2016)	Marchetto et al. (2010)
Fragile X syndrome	*FMR1*	Neural precursor cells Neurons (forebrain) Glial cells	Urbach et al. (2010), Sheridan et al. (2011), Doers et al. (2014) and Halevy et al. (2015)	Kaufmann et al. (2015) and Kumari et al. (2015)	Park et al. (2015)
Microcephaly	*ERCC6* *CDK5RAP2*	Neurons Cerebral organoids	Lancaster et al. (2013) and Vessoni et al. (2016)
Angelman/Prader-Willi syndromes	*UBE3A*	Neurons Astrocytes	Chamberlain et al. (2010)
Timothy syndrome	*CACNA1C*	Neural progenitor cells Neurons	Krey et al. (2013) and Tian et al. (2014)	Paşca et al. (2011)
Phelan-McDermid syndrome	Chromosome 22q13 deletion	Neurons (forebrain)		Shcheglovitov et al. (2013)
**Epilepsy**
Dravet syndrome	*SCN1A*	Neurons (dopaminergic, GABAergic) Forebrain interneurons Glutamatergic neurons Glial cells	Higurashi et al. (2013), Jiao et al. (2013), Liu et al. (2013, 2016) and Maeda et al. (2016)	Jiao et al. (2013)
Early infantile epileptic encephalopathy	*STXBP1*	Neurons (glutamatergic, GABAergic)	Yamashita et al. (2016)

**Movement disorders**
Hereditary spastic paraplegia	*SPG11* *ATL1* *SPAST*	Cortical neural progenitor cells Neurons (forebrain glutamatergic)	Denton et al. (2014); Havlicek et al. (2014) and Mishra et al. (2016)	Zhu et al. (2014)
*Ataxia telangiectasia*	*ATM*	Neural progenitor cells Neurons (GABAergic)	Nayler et al. (2012) and Carlessi et al. (2014)	Lee et al. (2013)
Friedrich’s ataxia	*FXN*	Neural progenitor cells Neural crest cells Neurons (peripheral sensory) Glial cells	Liu et al. (2011), Eigentler et al. (2013), Hick et al. (2013) and Bird et al. (2014)	Shan et al. (2014), Soragni et al. (2014) and Igoillo-Esteve et al. (2015)	Li et al. (2015)
Huntington’s disease	*HTT*	Striatal neural precursor cells Neurons (GABAergic striatal) Astrocytes	Camnasio et al. (2012), Chae et al. (2012), HD iPSC Consortium (2012), Jeon et al. (2012), Juopperi et al. (2012), Mattis et al. (2015) and Szlachcic et al. (2015)	Guo et al. (2013), Hsiao et al. (2014) and Lu et al. (2014)	An et al. (2012) and Cheng et al. (2013)
**Metabolic disorders**
Lesch-Nyhan syndrome	*HPRT*	Neurons	Mastrangelo et al. (2012) and Mekhoubad et al. (2012)
Niemann-Pick type C disease	*NPC1*	Neurons Astrocytes	Trilck et al. (2013, 2016)	Efthymiou et al. (2015)
Neuronal ceroid lipofuscinosis disease	*TPP1* *CLN3*	Neurons	Lojewski et al. (2014)
Gaucher’s disease	*GBA1*	Neurons (dopaminergic)	Awad et al. (2015) and Sun et al. (2015)	Tiscornia et al. (2013)
Metachromatic leukodystrophy	*ARSA*	Neural stem cells Astroglial progenitor cells	Doerr et al. (2015)
X-linked Adrenoleukodystrophy	*ABCD1*	Neurons Astrocytes Oligodendrocytes	Jang et al. (2011) and Baarine et al. (2015)
**Neuromuscular disorders**
Spinal muscular atrophy	*SMN1*	Neurons (motor neurons, forebrain neurons, sensory neurons) Astrocytes	Ebert et al. (2009), Chang et al. (2011), McGivern et al. (2013), Schwab and Ebert (2014), Boza-Morán et al. (2015), Demestre et al. (2015), Liu et al. (2015), Ng et al. (2015), Fuller et al. (2016), Heesen et al. (2016) and Patitucci and Ebert (2016)	Sareen et al. (2012), Ohuchi et al. (2016) and Xu et al. (2016)	Corti et al. (2012), Nizzardo et al. (2015) and Yoshida et al. (2015)

## Neural Differentiation of Human iPSC: A Wide Variety of Cell Types

Differentiation of iPSCs into neural cells is based on recapitulating embryonic development and relies on the use of specific factors that can promote or inhibit specific signaling pathways. All methods published so far can guarantee high purity of NSCs, but it is more challenging to obtain decent percentages of specific subtypes of desired mature neural cells. To date, it is possible to derive a wide variety of neuronal cell types from PSCs, including forebrain neuronal neurons (Espuny-Camacho et al., 2013; Lancaster et al., 2013); motor neurons (Wada et al., 2009; Nizzardo et al., 2010); dopaminergic neurons (Kriks et al., 2011; Kirkeby et al., 2012); GABAergic neurons (Maroof et al., 2013; Nicholas et al., 2013); medium spiny neurons (Delli Carri et al., 2013); forebrain cholinergic neurons (Wicklund et al., 2010; Hu et al., 2016); serotonergic neurons (Erceg et al., 2008); caudal neurons (Kirkeby et al., 2012); cerebellar neurons (Erceg et al., 2010); astrocytes (Emdad et al., 2012; Juopperi et al., 2012); oligodendrocytes (Nistor et al., 2005; Ogawa et al., 2011). Neuronal populations generated are typically heterogeneous, presenting both mature and immature cells, and thus need further technologies to achieve a high level of purity. Sorting techniques are often useful, but there are few neuronal subtype-specific surface markers available to select desired neural subpopulations (Pruszak et al., 2009; Yuan et al., 2011; Doi et al., 2014). To overcome this issue, sorting can sometimes be achieved by the expression of a selectable marker included as a reporter under expression of specific transcription factors or proteins (DeRosa et al., 2015; Toli et al., 2015).

## *In Vitro* Derived Neural Cells: What is their True Developmental Stage?

During reprogramming into iPSCs, somatic cells return to a developmental stage similar to that of ESCs, independent of their original age. Indeed, age-related characteristics of the original cells, (such as nuclear abnormalities, telomere length, and mitochondrial activity) are lost during this re-set to an embryonic stage (Marion et al., 2009; Suhr et al., 2010). Differentiation protocols for the generation of neuronal subtypes from PSCs require a significant amount of time spanning from weeks to months (Srikanth and Young-Pearse, 2014) to produce neurons that show a relatively mature morphological, molecular and electrophysiological phenotype. Despite this maturation process, generated neurons are still reminiscent of human fetal neurons (Mariani et al., 2012; Lancaster et al., 2013; Miller et al., 2013; Vera and Studer, 2015). It is therefore conceivable that such *in vitro* model systems could fail to recapitulate the disease phenotype especially for late-onset disorders.

Studer and colleagues (Miller et al., 2013) have addressed this issue, by developing a genetic strategy for introducing aging-related features in iPSC-derived neurons, specifically studying Parkinson disease (PD). Specifically, PD patient iPSC-derived midbrain dopaminergic neurons (mDA) recapitulate some PD disease features, including α-synuclein (αSYN) accumulation, oxidative stress, defects in neural outgrowth and mitochondrial dysfunction (Byers et al., 2011; Jiang et al., 2012; Reinhardt et al., 2013). However key late disease features of PD, such as neural degeneration, were only evident in model systems exposed to external stressors (Byers et al., 2011; Nguyen et al., 2011). Importantly, the accumulation of Lewy bodies and appearance of neuromelanin, a distinct feature of adult mDA neurons, have not been observed in iPSC-derived neurons. In this case, Studer and colleagues transiently overexpressed progerin, and showed restoration of aging features in both fibroblasts and mDA neurons derived from iPSCs. In particular, in iPSC-derived mDA neurons, they observed features of normal neural aging, with degeneration of dendrites *in vitro* and neuromelanin accumulation after grafting *in vivo* (Miller et al., 2013).

The relatively immature characteristics of iPSC-derived neurons should therefore always be a major consideration when modeling postnatal-, childhood- and adult-onset neurological diseases, where pathological features only manifest during postnatal development or the aging process.

## Variable Genetic Background: Defining Good Controls

Using patient derived iPSCs guarantees a unique opportunity to study the phenotype associated with a specific mutation in the context of the genetic background in which the mutation leads to disease. However, genetic background and potential genetic modulators of disease could conceivably affect the phenotype of both healthy control and patient lines. This is a definite limiting factor in the study of both Mendelian and more complex multigenic/multifactorial disorders. One solution is to use iPSCs derived from un-affected relatives for comparison, or to compare several control and several different patient lines in the same study. However, both these methods can be costly and time consuming. In more recent times, gene-editing technologies have become a more robust method by which the effect of a specific genotype on the iPSC model system can be unequivocally validated. Indeed, for monogenic disorders, correction of the single mutation in an iPSC cell line allows development of a unique “isogenic” control which harbors the same genetic background of the patient, thereby decreasing any “background noise” that could mask or affect cell phenotype. Furthermore, the insertion of a specific mutation into a control line can be utilized to show that such mutagenesis can induce disease phenotypes into a control line, akin to those seen in patient lines. Such concepts have been elegantly illustrated by Reinhardt et al. (2013) in their study of *LRRK2-*related PD, where they generated and compared several control lines, from healthy age- and gender-matched individuals, vs. isogenic controls using gene editing tools. No significant difference in αSYN levels was observed when comparing mDA neurons from wild type to *LRRK2*-mutated patient derived iPSC lines. However, αSYN levels were markedly reduced in patient lines when compared to the corresponding corrected isogenic lines. Gene expression profiles after 30 days of differentiation revealed significant differences in the age- and gender-matched iPSC lines when compared to both patient and isogenic lines. It is therefore clear that genetic background can have effects on gene expression, and the comparison of patient lines with isogenic controls can help overcome such genetic variability.

A number of different techniques can be used to generate isogenic controls. Reinhardt et al. (2013) used Zinc Finger Nuclease (ZFNs) technology. Both ZFN and the similar Transcription Activator-Like Effector Nuclease (TALENs) have been used successfully for gene editing in iPSCs (Hockemeyer et al., 2009, 2011). Both methods rely on the generation of costumed DNA binding domains conjugated to Fok1 nuclease, which can induce double strand breaks in a non-specific manner. The landmark discovery of the Clustered Regularly Interspaced Short Palindromic Repeats (CRISPR)-Cas9 system has added a highly efficient tool to manipulate the iPSCs genome (Ding et al., 2013) using endonuclease Cas9 and a guide RNA, targeting a specific region. Due to the efficient, relatively fast and targeted approach of the CRISPR-Cas9 technique compared to ZFNs and TALENs, this newer technique appears to be the preferred genome editing methodology for many researchers. Furthermore, the different commercial companies now offer generation of custom isogenic control lines using CRISPR-Cas9 technology (Baker, 2014).

## Neuronal Cells in a Dish: The Need for a 3D System

Although iPSCs represent an advantageous tool to study molecular phenotypes in neurological disease, their two dimensional nature means that some of the environmental factors, regional identities and complex neural circuits are absent when compared to either an animal disease model or human patient. Different studies have shown the intrinsic ability of NSCs to spontaneously self-organize in 3-dimensional (3D) structures resembling whole organs (Lancaster and Knoblich, 2014). Lancaster et al. (2013) showed how simple cultivation of control iPSCs in suspension can give rise to organoids which display several brain regions along the rostro-caudal and dorso-ventral pathways of mainly the forebrain and mid-hindbrain areas. The degree of cellular organization was maximal in the dorsal cortical region of the generated organoid where they observed a layered organization typical of the developing human forebrain. The same group then generated organoids from iPSCs derived from patients with microcephaly. The patient-derived cortical organoids mimicked a number of the features seen in disease, including smaller neural tissues with few progenitors regions, and radial glial maturation and orientation abnormalities, which were not previously recapitulated in a murine model. Organoids therefore represent a powerful tool to study both human fetal neocortical development (Camp et al., 2015) and developmental disorders. The generation of organoids representing more caudal fetal regions, (such as the midbrain and hindbrain) is also no doubt useful for studying neurological disorders affecting these areas of the central nervous system. Indeed, midbrain-like organoids (MLOs) have recently been derived from human ESCs (Jo et al., 2016). Neural cells of such MLOs not only expressed midbrain markers and displayed characteristic dopaminergic electrical activity, but also produced neuromelanin, a characteristic not observed in bi-dimensional systems.

## Moving iPSCs into Clinical Utility: Drugs, Cell and Gene Therapy for Pharmacoresistant Childhood Neurological Disorders

The development of new drugs to treat human disorders is a challenging field. Indeed, many drugs tested in animal models have failed in human clinical trials due to lack of efficacy or intolerability (Scannell et al., 2012). Overall, new drugs for pharmacoresistant disorders are an unmet need, and constitute a priority area for research. The development of new drugs is hindered by the lack of appropriate models. Human iPSCs offer a unique opportunity for high-throughput drug screening in patient derived cells to assess drug efficacy and toxicity. Despite being in the early stages, several studies have demonstrated the feasibility and usefulness of this approach for childhood neurological disorders. For example, motor neurons derived from patients affected by Spinal Muscular Atrophy (SMA) have been used to test specific drugs (Xu et al., 2016). SMA is characterized by mutations in *SMN1*, which leads to degeneration of spinal motor neurons associated with mitochondrial dysfunction. Treatment of SMA human iPSCs derived motor neurons with N-acetylcysteine (NAC) improved mitochondrial functionality, thereby rescuing motor neuron degeneration *in vitro*.

In Rett syndrome (RTT) patient-derived iPSCs have been utilized to test the effect of IGF1 and gentamicin *in vitro* (Marchetto et al., 2010). RTT iPSCs derived glutamatergic neurons showed a decreased level of glutamatergic synapses when compared to controls, which was increased by IGF1 treatment. Gentamicin, administrated at high dose acted as a suppressor of nonsense mutations which cause impaired function of MeCP2 in RTT. Furthermore, IGF1 has been used to rescue the phenotype observed in neurons differentiated from Phelan–McDermid syndrome (PMDS)-derived iPSCs (Shcheglovitov et al., 2013). PMDS neurons showed impairment in excitatory synaptic transmission and reduced number of excitatory synapses, which were restored after treatment with IGF1.

These and other studies (Table 1), have mainly tested small number of compounds on iPSC differentiated cells, thereby demonstrating the feasibility of using iPSCs for drug testing. In the future, high-throughput technologies allowing the screening of an extensive library of compounds will be useful. High-content imaging and analysis are likely to be helpful for such high-throughput approaches (Sirenko et al., 2014). High-content high-throughput assays have already been undertaken on iPSC-derived neural cells in 348-well assays, with analysis focusing on neurite outgrowth, cell number and viability, mitochondrial integrity and membrane potential.

Two studies have used high-throughput assays to screen large numbers of candidate drugs for Fragile X syndrome, a neurodevelopmental disorder characterized by learning problems, autism, and anxiety. This syndrome is associated with CGG repeat expansion in the 5′-untranslated region of *FMR1*, which leads to the absence of FMR protein. Both studies used patient derived iPSCs and differentiated them either in NSCs or neural precursors. In the first study 5000 compounds (both novel and approved drugs) were tested (Kumari et al., 2015), while the second study expanded drug screening to over 50,000 compounds (Kaufmann et al., 2015). In both studies FMR1-neural cells, treated with compounds as decanehydroxamate, deserpidine or tibrofan, showed increased mRNA levels, though not to clinically significant levels. Nevertheless, both studies show how promising high-throughput techniques can be in expanding the potential of drug testing using iPSCs derived cells.

In addition to the screening of new drugs, human iPSCs also offer other therapeutic possibilities that can be translated into clinical practice, as evident in the Phase I SMA study (Chiriboga et al., 2016). The antisense oligonucleotide nusinersen was designed to alter splicing of SMN2 mRNA. SMN1 motor neurons compensate with the paralogous gene *SMN2*. *SMN2* shows a high sequence homology to *SMN1*, the only difference resides in the C-to-T base change inside exon 7. This mutation leads to abnormal SMN2 splicing and to the generation of truncated highly unstable proteins that trigger neural degeneration of motor neurons. SMN2 splicing correction with the use of oligonucleotides resulted in the production of a greater amount of full-length SMN2. This strategy has been tested on patient-derived iPSCs differentiated motor neurons (Corti et al., 2012) and showed the ability to convert SMA-differentiated motor neurons to a normal phenotype, both *in vitro* and after grafting into a mouse model of disease.

iPSC-derived neural cells can also be a patient-derived platform to test genetic therapies. Juvenile neuronal ceroid lipofuscinosis (NCL) disorder is caused by loss-of-function mutations in *CLN3*. Patient-derived iPSCs neurons showed abnormal lysosomal storage with abnormalities observed in mitochondria, Golgi apparatus and endoplasmic reticulum. After restoring the function of CLN3 via AAVrh.10 virus bearing wild-type human *CLN3*, *in vitro* differentiated neurons showed a rescued phenotype, without excess accumulation of storage material (Lojewski et al., 2014). Similarly, a lentiviral approach was used for a genetic early onset form of PD. iPSC-derived mDA harboring mutations in *PINK1*, a gene encoding a mitochondrial kinase, showed dysfunctional mitochondrial function. Expression of the non-mutated PINK1 via lentivirus in patient-derived mDA neurons restored normal recruitment of PINK1 upon mitochondrial depolarization, and normalized mitochondrial number and biogenesis (Seibler et al., 2011).

The combination of genetic engineering techniques and the promise of human PSCs differentiated cells as a donor source for cell replacement therapies, could in the future lead to the generation of patient-derived “corrected” cells that could potentially be used in autologous transplantation to replace affected disease cells. Such use of patient derived, genetically corrected neural cells may also potentially overcome immune-mediated responses that might be triggered by using allogenic neural cells. Overall, even though such approaches are extremely promising and although PSCs are already used in clinical trials (Kimbrel and Lanza, 2015), there remain many issues regarding the use of PSC as cell replacement therapy, particularly concerning cell identity, purity, safety and long term risks. For this reason, even though it remains an extremely promising approach, clinical translation of such a therapeutic approach is likely to take some time.

## Conclusion

Patient derived iPSCs represent a unique and increasingly utilized tool for the study of human genetic neurological diseases of childhood. iPSCs are an extraordinary model that can facilitate new insight into the molecular basis of disease and aid the development of new therapies, especially for pharmacoresistant diseases where human tissue is inaccessible for research purposes. Like all other laboratory models, human iPSCs have some limitations, namely that the model can be time consuming and costly to establish, shows clonal variability and genetic background can influence phenotype. Despite this, the ever-growing number of studies using human iPSCs to both model genetic disease and discover new therapies, render them an extremely promising tool, capturing the attention of researchers worldwide.

## Author Contributions

SB: conceptional design and writing of the manuscript. MAK: conceptional design and writing of the manuscript.

## Conflict of Interest Statement

The authors declare that the research was conducted in the absence of any commercial or financial relationships that could be construed as a potential conflict of interest. The handling Editor declared a shared affiliation, though no other collaboration, with the authors SB, MAK and states that the process nevertheless met the standards of a fair and objective review.
